# Protocol for a randomized comparison of integrated versus consecutive dual task practice in Parkinson’s disease: the DUALITY trial

**DOI:** 10.1186/1471-2377-14-61

**Published:** 2014-03-27

**Authors:** Carolien Strouwen, Esther ALM Molenaar, Samyra HJ Keus, Liesbeth Münks, Marten Munneke, Wim Vandenberghe, Bastiaan R Bloem, Alice Nieuwboer

**Affiliations:** 1 Department of Rehabilitation Sciences, KU Leuven, Faculty of Kinesiology and Rehabilitation, Tervuursevest 101 bus 1501, Leuven 3001, Belgium; 2 Department of Neurology, Radboud university medical centre, Nijmegen Centre for Evidence Based Practice, Nijmegen, The Netherlands; 3 Department of Neurology, University Hospitals Leuven, Leuven, Belgium; 4 Department of Neurosciences, KU Leuven, Leuven, Belgium; 5 Department of Neurology, Radboud university medical centre, Donders Institute for Brain, Cognition and Behaviour, Nijmegen, The Netherlands

**Keywords:** Parkinson disease, Rehabilitation, Physical therapy, Neurologic gait disorder, Cognition, Dual task

## Abstract

**Background:**

Multiple tasking is an integral part of daily mobility. Patients with Parkinson’s disease have dual tasking difficulties due to their combined motor and cognitive deficits. Two contrasting physiotherapy interventions have been proposed to alleviate dual tasking difficulties: either to discourage simultaneous execution of dual tasks (consecutive training); or to practice their concurrent use (integrated training). It is currently unclear which of these training methods should be adopted to achieve safe and consolidated dual task performance in daily life. Therefore, the proposed randomized controlled trial will compare the effects of integrated versus consecutive training of dual tasking (tested by combining walking with cognitive exercises).

**Methods and design:**

Hundred and twenty patients with Parkinson’s disease will be recruited to participate in this multi-centered, single blind, randomized controlled trial. Patients in Hoehn & Yahr stage II-III, with or without freezing of gait, and who report dual task difficulties will be included. All patients will undergo a six-week control period without intervention after which they will be randomized to integrated or consecutive task practice. Training will consist of standardized walking and cognitive exercises delivered at home four times a week during six weeks. Treatment is guided by a physiotherapist twice a week and consists of two sessions of self-practice using an MP3 player. Blinded testers will assess patients before and after the control period, after the intervention period and after a 12-week follow-up period. The primary outcome measure is dual task gait velocity, i.e. walking combined with a novel untrained cognitive task to evaluate the consolidation of learning. Secondary outcomes include several single and dual task gait and cognitive measures, functional outcomes and a quality of life scale. Falling will be recorded as a possible adverse event using a weekly phone call for the entire study period.

**Discussion:**

This randomized study will evaluate the effectiveness and safety of integrated versus consecutive task training in patients with Parkinson’s disease. The study will also highlight whether dual task gait training leads to robust motor learning effects, and whether these can be retained and carried-over to untrained dual tasks and functional mobility.

**Trial registration:**

Clinicaltrials.gov NCT01375413.

## Background

Parkinson’s disease (PD) is a common, multisystem neurodegenerative disease which is characterized by motor and non-motor symptoms [1]. The motor symptoms of PD are manifold but include gait and balance disorders, which have a significant impact on functional mobility and quality of life [2]. Depending on the disease stage, up to 79.2 percent of patients (advanced stage) may report to have freezing of gait (FOG) [3], which is a disabling gait disorder characterized by episodes of lack of forward progression despite the intention to walk [4]. Approximately 60% of patients fall each year and about two thirds fall recurrently [5-7].

Dual tasking is the simultaneous performance of two attention-demanding tasks with different goals, whereby one task can be denoted as the primary and the other as the secondary task [8]. Factors that affect dual task performance are the environment in which the task takes place, the nature of the secondary task, the age and disease-specific factors of each individual [9]. Gait has been found to deteriorate during dual task (DT) performance in PD [10-14], resulting in a decrease of gait velocity, cadence and step length [9,13-18], an increase in gait variability [9,13,17,19] and an increase in double support time [14,20]. Also, falling and FOG are more commonly provoked in DT conditions [11,21,22].

Mild cognitive impairment is thought to occur in 20-57% of PD patients, even as early as 3–5 years after the diagnosis [23,24]. Several studies have indicated that executive dysfunction is a robust determinant of DT interference at least in mid but not in early stage PD [13,17,25,26]. Executive function refers to a set of abilities which flexibly guide behavior towards goals and includes switching between cognitive sets or tasks, appropriately inhibiting and generating responses and updating working memory contents [27,28]. Wild et al. [13] showed that cognitive performance in PD worsened during DT walking and that this was correlated to global cognitive condition.

Motor learning is highly dependent on cognitive status in PD [29,30]. Patients with freezing of gait (FOG) showed greater executive deficits than their non-freezing counterparts [31-33] and were also found to learn a serial reaction time task less well [34]. This raises the question as to the extent of the cognitive challenge which is appropriate for achieving robust learning in PD.

Evidence for the efficacy of physiotherapy is growing in PD [35,36]. Various modes of gait training were found effective in improving gait velocity [37,38]. Although more controlled studies remain needed, cognitive training shows promising benefits in several cognitive domains including executive function in PD [39]. Despite the fact that evidence-based physiotherapy guidelines discourage the use of dual task exercise [40], a number of open label studies [38,41-46] support the feasibility and efficacy of DT training in PD [45,46]. Hence, a phase III randomized study is currently being undertaken in which the effect of single and dual task gait training is compared in a wide variety of PD patients [47].

The current trial proposes to test the efficacy of two strategies for DT training. The first strategy entails consecutive task training (CTT), whereby each task will be trained separately. We expect that as a result of this type of training, performance of each task may become more automatic and thus free residual brain capacity for subsequent simultaneous task performance. The second strategy proposes integrated dual task practice (IDT). We expect that this training mode may increase the efficiency of shared neural resources [48] and as such improve DT performance. IDT may have additional benefits over CTT as this training mode may also improve the efficiency of specific brain areas involved in task integration [49,50], enhance executive function and ease the transfer of learning to daily DT situations. Moreover, an advantage of IDT is that dual tasking can realistically never be avoided entirely, so it might be wise to prepare patients for such inevitable dual task events that commonly occur in daily life. Interestingly, in balance-impaired older adults, IDT and CTT led to largely similar performance increases with the exception of better retention in IDT [51,52]. These findings call for replication, and importantly, it is currently unknown to what extent these results apply to PD patients.

Therefore, the primary aim of this study is to compare the effectiveness of consecutive versus integrated training (tested by combining walking with cognitive exercises) on DT gait performance in PD. We hypothesize that IDT practice will resort in better dual task outcomes and better consolidated learning results, particularly in patients without cognitive impairment. As secondary questions we will examine which dual task training mode is most beneficial in terms of fall risk reduction and leads to the best dual task gains in freezers versus non-freezers. Here, we describe the design of this training study, also referred to as the DUALITY trial.

## Methods/design

### Study design and setting

**Figure 1:**
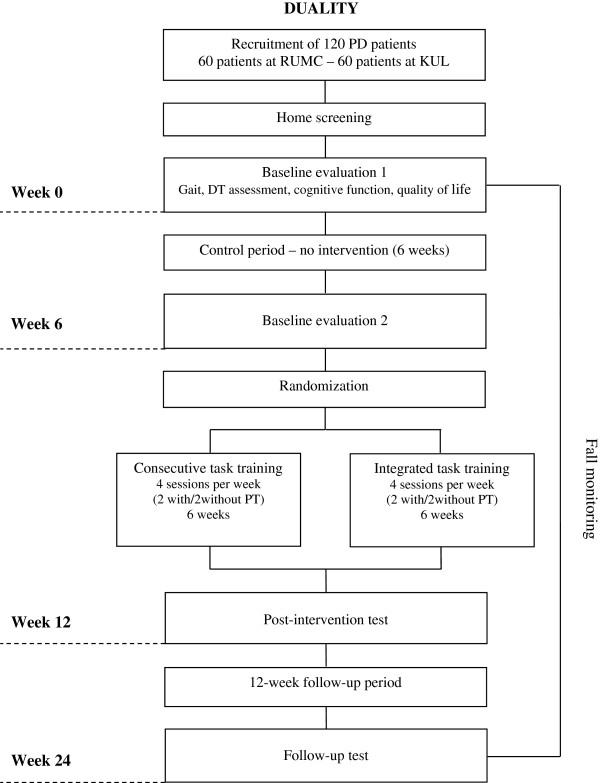
**Design of the Duality study.** PD: Parkinson’s disease. RUMC: Radboud University Nijmegen Medical Centre. KUL: Catholic University Leuven. DT: dual task. PT: physiotherapist.

The study has a parallel group design and involves a dual-centered, single blind, randomized controlled trial with a 12 week follow-up period. The study will include 120 patients with PD of Hoehn & Yahr stage II to III [53]. Individuals with PD will be randomly allocated to two arms: (a) six weeks of integrated dual task training (IDT); or (b) six weeks of consecutive task training (CTT). Both interventions will be delivered at the patient’s home with the same frequency and intensity: 12 supervised sessions by a physiotherapist and 12 unsupervised training sessions. Preceding the intervention, participants will undergo a six week control period without training to ascertain the effect of repeated measures (Figure 1). All other interventions (medication, allied health care) will be kept as stable as possible. Any changes in treatment or medication will be monitored. The trial will be conducted in two countries. Sixty patients will be recruited at the Radboud University Medical Center (RUMC) in the Netherlands and sixty patients will be recruited at the University Hospitals in Belgium.

### Participants

Inclusion criteria for recruitment are: (a) diagnosis of PD according to the UK Brain Bank criteria [54]; (b) Hoehn and Yahr stage II-III in the subjective best ON-phase of the medication cycle [53]; (c) able to walk 10 minutes continuously; (d) presence of dual task interference as established by a structured checklist (see Table 1); (e) a score ≥ 24 on the Mini Mental State Examination (MMSE) [55]; (f) stable medication over the past 3 months; (g) no hearing and visual problems that interfere with testing or training; and (h) stable Deep Brain Stimulator (DBS) settings over the past year. Exclusion criteria are: (a) unstable medical conditions including acute orthopedic conditions affecting gait; and (b) ongoing dual task training or other interfering physical therapy. Patients who received dual task training in the past are allowed to participate if at the moment of inclusion they report to have dual task problems based on a structured checklist (Table 1). Recruitment of patients will be conducted via the databases of the Movement Disorder clinic at University Hospitals Leuven and RUMC and affiliated medical centers. All interested participants will be screened by a physiotherapist during a first home visit in which informed consent will be obtained and inclusion criteria applied. Full ethical approval has been granted for the study in the Netherlands (CMO Regio Arnhem-Nijmegen) protocol ID/number NL39530.091.12 and in Belgium (CME KU Leuven) B322201213165/S53419.

**Table 1:** Dual task screening list to determine dual task problems

**Do you experience difficulties with the combination of …**	**Yes**	**No**	**Not applicable**
Walking and talking			
Walking and phoning			
Walking and carrying a bag			
Walking and carrying a plate filled with food			
Walking and carrying a filled glass			
Walking and avoiding obstacles			
Walking and getting something out of your pockets (tissue, money, mobile phone)			
Walking outside and paying attention to traffic			
Walking and remembering things (phone number, address)			
Walking and thinking about something else			
Walking and looking for items while shopping			
Walking and closing the zipper of your jacket			
Walking and finding your way in airport or train station			

### Sample size

Primary outcome is DT gait velocity. Single task gait velocity changes of around 0.05 m/s have been found clinically meaningful [38] but no such data presently exist for dual task gait velocity. Sample size power calculation [56] was based on data from the RESCUE trial in which in a similar study population had a mean dual task gait speed of 0.77 m/s (SD = 0.21) [38,57]. Power was set at 80% and based on two-sided 95% confidence intervals. We assumed that there would be a difference of 15% between both arms in favor of IDT based on previous study [51]. In analogy to the RESCUE study, in which a home-based intervention was delivered, a drop-out rate of 5% was envisaged. Assuming a stable control period, we calculated our sample size to include a total of 108 subjects (54 per center). Incorporating a loss to follow-up, we will aim to recruit 60 patients (total of 120 subjects) per group over a period of two years.

### Randomization and blinding

Subjects will be randomly assigned per center to the earlier described integrated dual task (IDT) or consecutive task training (CTT). A computerized block randomization procedure will be implemented by an independent statistician using a block size of four subjects. Group allocation will be performed by an independent person, who will notify the treating physiotherapist by email to ensure concealed allocation. Randomization will be stratified by subgroup with or without FOG and by stage of the disease (Hoehn & Yahr stage II and III). To avoid bias, patients are assessed by blinded testers. In addition, participants will be explicitly instructed not to reveal any details about their training regime during testing to prevent unblinding. Both therapists and patients will be explained that both arms of the study are likely to be effective in improving dual task performance to control for expectancy effects, in line with the above outlined rationale for each training approach.

### Intervention

**Figure 2:**
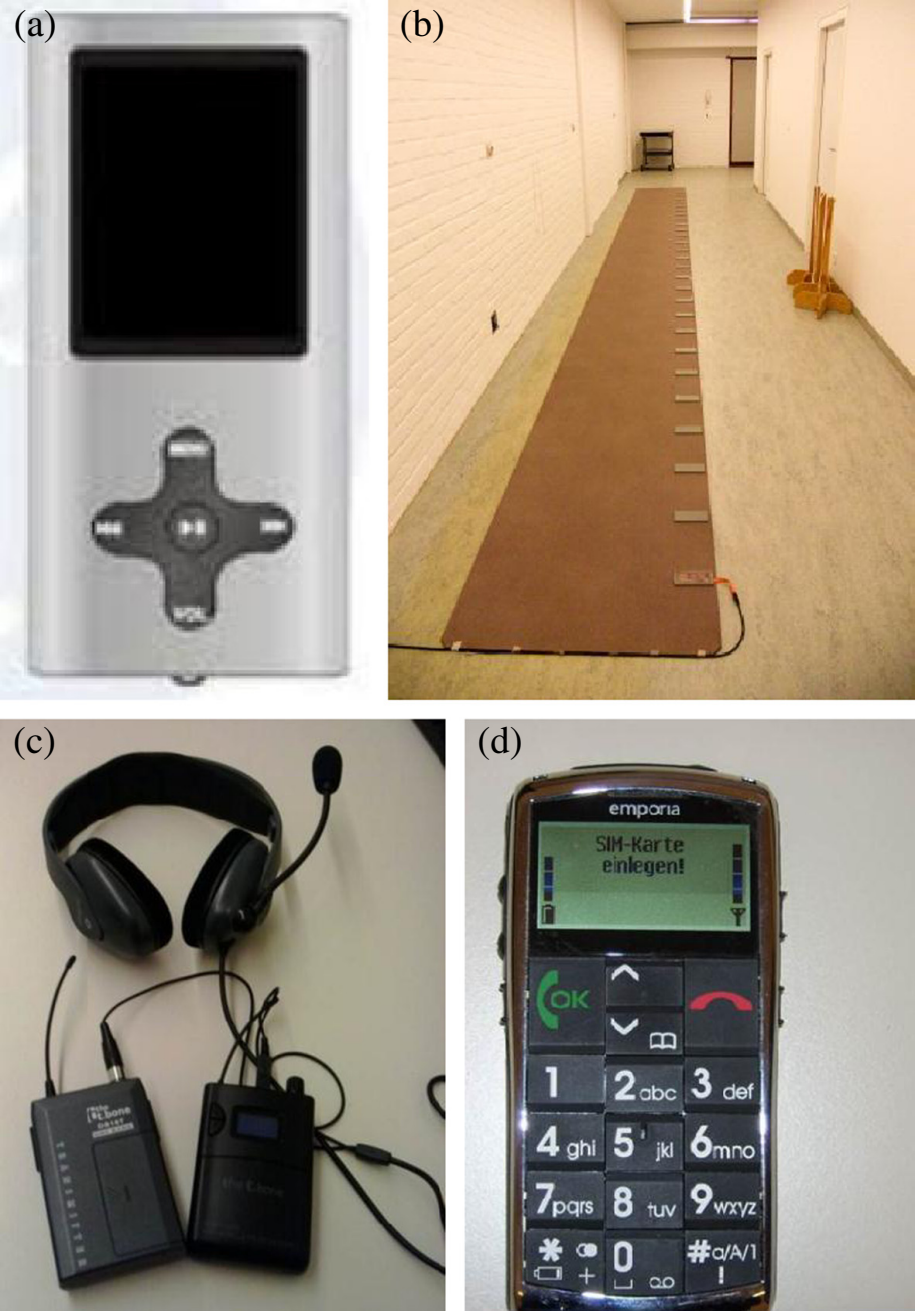
**Equipment used during training and testing. (a)** MP3-player (DIFRNCE MP1850); **(b)** GaitRite Electronic Walkway System; **(c)** wireless headset system (Beyerdynamic; transmitter: t-bone DS16T and receiver: t-bone IEM100R); **(d)** large buttoned mobile phone (EmporiaTalkPremium).

Both interventions are delivered by trained physiotherapists, twice a week at the patient’s home. These sessions entail 30 minutes of supervised gait and cognitive exercises and 10 minutes of functional practice. Unsupervised exercises will be conducted twice a week for 30 minutes and include gait and mental practice using an MP3 player. MP3-player delivery of cognitive training was found feasible in an earlier pilot DT study [45]. User-friendly devices with a large display and buttons (DIFRNCE MP1850) will be used (Figure 2a). Therapists will assess whether the home exercise is perceived as safe and without risk of falling or needs to be performed together with a carer.

In order to contrast CTT and IDT training precisely and ensure that both training arms offered sufficient challenge for a variety of patients, a standardized program of cognitive training based on structured progression levels was developed by an expert team of four physiotherapists with the input of 1 psychologist. Hence, the programs for CTT and IDT consist of three identical components: (1) gait practice; (2) auditory cognitive exercise; and (3) functional training suitable to be delivered at home (Table 2). The program was piloted on several patients at home in the first six months of trial preparation.

**Table 2:** The duality training program

**Intensity**			
6 weeks	2x/week	40 min	Therapy session with physiotherapist
	2x/week	30 min	Therapy session without physiotherapist
**Integrated training group**			**Consecutive training group**
- Gait training while doing cognitive exercises			- Cognitive training while sitting
- Functional training of dual tasks in ADL			- Gait training without extra cognitive load
			- Consecutive functional training avoiding dual tasks
**Contents of components in both training arms**			
	**Exercises**		**Progression**
**Gait training**	Depending on clinical need walking while:		Increase speed
	Focussing on big steps		Increase amplitude
	Focussing on heel strike		Increase or decrease frequency
	Focussing on arm swing		Introduce speed variations
	Focussing on upright posture		
	Walking while raising knees		Decrease exercise execution time
	Tandem gait		Increase coordination demands
	Turning		Increase environmental demands: surfaces, narrow spaces, doorways, outdoors
	Transitions: sit-to-stand, start-stop		
	Stepping in multi-directions		
**Cognitive training**	*Verbal fluency tasks*		
	e.g. Name cities and countries starting with A,B,C		More difficult categories
	*Discrimination and decision making tasks*		
	e.g. Say yes when you hear “strawberry” but say nothing when you hear another sort of fruit		Decrease response time, responding to two or three different words
	*Working memory tasks*		
	e.g. Digit span backwards, Word memory task		Increase the length of series, related vs. unrelated words
	*Mental tracking tasks*		
	e.g. Count how many times you hear the word “cat” in this story about cats.		Count two or three words in a story
	Counting: summing and subtracting		Increase the complexity of the counting task
	*Reaction time tasks*		
	e.g. React as fast as you can on a certain word or sound		Decrease time between two reactions, react to two or three words or sounds
	Cognitive exercise is delivered by the therapist using a laptop.		
	During self-administered practice, cognitive exercise is played via an MP3 player		
**Functional training**	*Integrated training group*		
	Walking while having a conversation		
	Describing route while walking		
	Taking wallet, handkerchief, … out pocket while walking		
	Closing buttons and zipper while walking		
	Carrying tray, cup, plate		
	Carrying groceries		
	Moving kitchenware from sink to cupboard		
	*Integrated AND consecutive training group*		
	Laying the table		
	Picking object from the ground		
	Maneuvering in bathroom		
	Getting mail out the mailbox		
	Self-reported functional difficulties		

Table 2 illustrates the components of training in both study arms. Gait practice involves specific gait exercises aimed to improve gait quality at home. Progression is introduced once patients can perform the exercise fluently (CTT) or without noticeable DT interference (IDT). Cognitive exercises (Table 2) are offered in five categories demonstrated to cause gait interference in older people [58]: (1) verbal fluency; (2) discrimination and decision making tasks; (3) working memory tasks; (4) mental tracking tasks; and (5) reaction time tasks. The cognitive exercises and levels of progression are audiotaped, allowing segments to be played on the therapists’ laptops or on the MP3 players for self-practice. Subjects will be instructed to respond to the tasks with spoken word sequences. Verbal responses are monitored and scored by the therapist, who provides feedback on performance after each bout of practice. Flawless performance at the starting level (CTT) or no noticeable interference during DT (IDT) will be adopted as guidelines to progress to the next level. Functional tasks, relevant for each patient, are chosen for the functional part of the training program to ensure generalization of practice (Table 2).

Table 2 also shows that in the CTT arm of the study, each session will consist of 15 minutes of gait practice, 15 minutes of cognitive practice and 10 minutes of functional task training. Gait practice is focused on improving gait quality. Cognitive training is performed while the patient is sitting on a chair. CTT functional training will emphasize safety, avoiding dual tasking and carrying out task components separately as much as possible. Table 2 also shows that in contrast IDT is based on performing motor-cognitive tasks concurrently for 30 minutes right from the first session. Gait practice is performed while at the same time verbally responding to the cognitive exercises. Given the likely cognitive deterioration in PD [29,30], a fixed priority of dividing attention on both tasks will be implemented in the beginning of the training, aimed to improve better and safe walking. If possible a variable locus of attention [51,52] is adopted as the patient progresses. During functional task practice, dual tasking will be positively encouraged to ensure transfer of learning to daily life.

Rigorous measures of standardization of the interventions are implemented between the two centers by having a cross-center training week of therapists at the onset of the trial and by regular follow-up meetings.

### Testing and outcome measures

Table 3 gives an overview of the outcome measures which will be tested at various time points. The primary outcome measure is DT gait velocity while performing an untrained auditory Stroop task. The clinical test battery includes the following descriptive, disease and cognitive characteristics assessed at baseline: Dual Task screening questionnaire (Table 1); MMSE [55]; Montreal Cognitive Assessment (MoCA) [59,60]; Frontal Assessment Battery (FAB) [61]; Unified Parkinson’s Disease Rating Scale part I, II and IV; and retrospective fall frequency (past year). Following questionnaires are assessed at all four time points: the Unified Parkinson’s Disease Rating Scale part III (MDS UPDRS-III) [62]; the new Freezing of Gait Questionnaire (new FOGQ) [63]; the Activities specific Balance Confidence Scale(ABC-scale) [64]; the Scales for Outcomes of Parkinson’s disease-Cognition (ScopaCog) [65]; the Alternating Names Test(ANT)/Alternating Intake Test (AIT) [66] and the Parkinson’s disease Questionnaire for quality of life (PDQ-39) [67].

**Table 3:** Primary, secondary and tertiary outcome measures

	**Outcome measures**	**Baseline 1**	**Baseline 2**	**Post-intervention**	**12 week follow-up**
**Primary outcome measure**					
DT gait performance	Gait velocity Stroop	x	x	x	x
**Secondary outcome measures**					
DT gait performance	Cadence, Stride length Stroop	x	x	x	x
	Gait variability Stroop	x	x	x	x
	Gait velocity, Cadence, Stride length Digit	x	x	x	x
	Gait variability Digit	x	x	x	x
	Gait velocity, Cadence, Stride length Mobile	x	x	x	x
	Phone				
	Gait variability Mobile Phone	x	x	x	x
ST gait performance	Gait velocity	x	x	x	x
	Cadence	x	x	x	x
	Stride length	x	x	x	x
	Gait variability measures	x	x	x	x
DT cognitive tasks	Reaction time digit	x	x	x	x
	Response time digit	x	x	x	x
	Errors digit	x	x	x	x
	Reaction time Stroop	x	x	x	x
	Errors Stroop	x	x	x	x
	Errors Mobile phone	x	x	x	x
ST cognitive tasks	Reaction time digit	x	x	x	x
	Response time digit	x	x	x	x
	Errors digit	x	x	x	x
	Reaction time Stroop	x	x	x	x
	Errors Stroop	x	x	x	x
	Errors Mobile phone	x	x	x	x
**Tertiary outcome measures**					
Adverse effects	Number of falls	Weekly follow-up			
Motor function	MDS-UPDRS III	x	x	x	x
	New FOGQ	x	x	x	x
	ABC	x	x	x	x
Cognitive function	Scopa-Cog	x	x	x	x
	Alternating fluency (ANT/AIT)	x	x	x	x
	FAB	x	-	-	-
Descriptors	Disease duration	x	-	-	-
	Medication dose	x	x	x	x
	Hoehn and Yahr stage	x	-	-	-
	MMSE	x	-	-	-
	MoCA	x	-	-	-
Quality of life	PDQ-39	x	x	x	x
Patient experience	Focus interview	-	-	-	x

DT: Dual task. ST: Single task. MDS-UPDRS part III: Unified Parkinson’s Disease Rating Scale – motor part. H-Y: Hoehn and Yahr stage. New FOGQ: new Freezing of Gait Questionnaire. ABC: Activities specific Balance Confidence scale. Scopa-Cog: Scales for Outcomes in Parkinson’s disease – cognitive. ANT: Alternating Names Test. AIT: Alternating Intakes test. MMSE: Mini Mental State Examination. MoCA: Montreal Cognitive Assessment. FAB: Frontal Assessment Battery. PDQ-39: Parkinson’s Disease Quality of Life Questionnaire.

After the 12 weeks follow-up, a qualitative interview will be conducted probing patients’ perceptions about the intervention and how it affected their ADL-performance. All assessments will be performed in the ON-phase at a standardized moment after medication intake. Standardization of testing procedures between the two centers is applied by regular meetings and shared testing sessions. Any adverse effects will be recorded and the weekly number of falls will be determined.

The primary and secondary gait outcomes will be measured with the same GAITRite Walkway System embedded in the gait laboratories of both centers [68]. The order of the GAITRite testing procedures and the clinical test battery will be conducted randomly but will be kept constant in each patient. The GAITRite mat, placed in a quiet laboratory space, uses pressure sensors to detect footfalls during walking (see Figure 2b). The GAITRite has been found a reliable system for measuring spatiotemporal gait parameters over time [68]. Gait outcomes will be measured with and without secondary tasks at comfortable walking speed. An average of two trials per condition will be used for statistical analysis.

Performance on the secondary tasks will be assessed during walking as well as in sitting position. The order of single and dual task assessments is determined randomly and will remain the same in each test session for an individual patient. In the DT conditions, a verbal signal is given to the patient to start walking and at the same time the secondary task is started to synchronize the measurements.

Three secondary tasks are used to assess dual task performance: (a) an auditory Stroop task [69,70]; (b) a Backwards Digit Span task [71,72]; and (c) an especially designed functional mobile phone task (MPT). The auditory Stroop task is an untrained dual task and represents the primary outcome. During this task, the patient will verbally respond to congruent and incongruent high and low tones. The patient hears three different trials consisting of four stimuli. Stimuli are presented with a variable interval (1.5 – 2 seconds) to control for cueing effects. The difficulty level is the same for all participants. The Stroop task assesses set shifting ability and inhibition of incongruent responses. During the Backwards Digit Span task, the patient will have to repeat an array of numbers in reverse, the length of which will be adapted to the level of the patient and is determined at baseline. The Backwards Digit Span is a trained task and loads working memory, as part of executive function.

**Figure 3:**
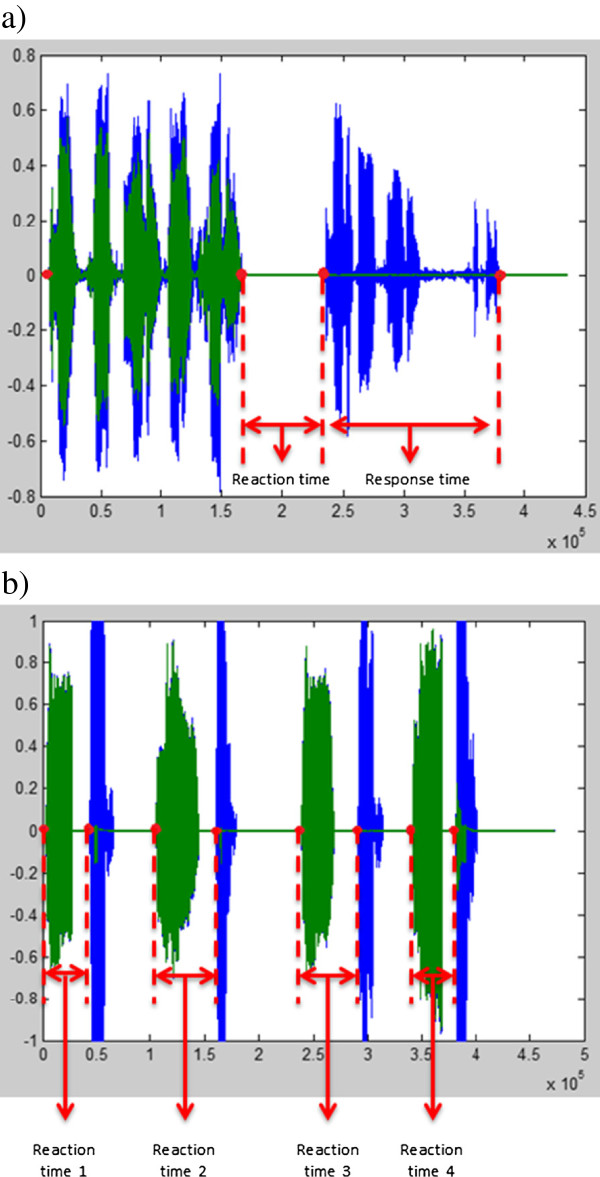
**Visual presentation of reaction and response times in Matlab R2011b. (a)** Visual presentation of Backwards Digit Span task – green = stimulus that is heard by the patient, blue = answer of the patient; **(b)** Visual presentation of Stroop task. Since four stimuli per fragment are given, four reaction times per fragment can be calculated – green = stimulus that is heard by the patient, blue = answer of the patient.

Verbal responses to both the Backwards Digit Span task and the Stroop task will be recorded via a wireless headset system (Beyerdynamic; transmitter: t-bone DS16T and receiver: t-bone IEM100R) (Figure 2c). Verbal responses are recorded and saved in the same channel as the sound fragments, which guarantees optimal synchronization, and measurement of the number of correct responses, reaction (Digit Span task and Stroop task) and response times (Digit Span task). Figure 3 indicates how reaction time is defined for the Stroop and Digit Span task. Response time is the total duration of verbalizing a backward sequence of the Digit Span task. Analysis of reaction and response times will be performed using Audacity 1.3 Beta program and Matlab (R2011b).

The custom-made mobile phone task is a combined motor and cognitive task, in which the patients will have to type the test date (8 numbers) into a large-buttoned mobile phone (Emporia Talk Premium; Austria) (see Figure 2d). The number of errors and the time it takes to complete this task will be measured. The task is an untrained DT and tests recall and working memory as well as fine motor skills.

### Falls monitoring

For the entire study period of 24 weeks, the patient will receive a weekly phone call to ask about any fall events in the previous week. In case of a fall, questions are asked about the consequences and specific circumstances in which the fall took place (Table 4). Individual fall records will be communicated to the therapist, to enable adaptation of treatment. If a worrying trend of an increased fall risk would occur as a result of the treatment, therapists are expected to adapt their treatment accordingly and report this to the safety board of the trial. In addition, therapists will record any falls that may occur during the intervention. If safety adaptations imply a reduction of the intensity of treatment or in essence will require a change from an integrated to a consecutive training, patients will be considered to become a dropout and are included for the intention to treat analysis. The review board of the trial will have a six-monthly meeting in which the global fall rates will be monitored and discussed. A-priori fall rate increases as cutoffs for trial cessation will not be determined as falling can be very variable in PD and fluctuate according to medication status.

**Table 4:** Weekly follow-up of falling

**In case of a fall:**	
**Description of fall**	When did you fall?
	At what time of the day?
	Can you describe what happened at the moment you fell?
	What were you doing?
	What was the cause of the fall?
	Where did you fall (inside or outside)?
**Medication**	When was the last time before the fall that you took medication?
	Was this medication still working (on or off)?
**Freezing**	Did freezing occur at the time of the fall?
**Dual tasking**	Were your hands free at the time of the fall?
	Were you talking to someone at the time of the fall?
**Adverse aspects related to fall**	Did you have any injury related to the fall?
	Are you more afraid of falling?

### Data analysis/statistics

A statistical analysis plan will be developed prior to unblinding and analysis. The primary data analysis will be performed according to an intention to treat principle. The primary endpoint, the DT walking performance at test session 1, 2, 3 and 4 will be evaluated using a linear mixed model. Tests will be two-sided with α 0.05. The fixed factors will be treatment (IDT vs. CTT), test session (1, 2, 3 and 4) and the interaction between test session and treatment group. Subject will be a random factor. The treatment difference will then be estimated by the appropriate contrast for the difference between the mean scores at test session 1, 2, 3 and 4. Secondary endpoints will be analyzed in a similar way. As a secondary question we will also model the three-way interaction between subgroup, i.e. those with and without freezing of gait (defined as a score of 1 on the New FOGQ), treatment group and time. Fall frequency data will be analyzed using a negative binomial model depending on the distribution of the data.

The influence of center, disease severity (Hoehn and Yahr scores) and cognition will be explored by including the interaction terms between treatment and each of these variables in the model. Throughout, 95% confidence intervals will be determined. Sensitivity analyses will be carried out to evaluate the impact of missing values on the outcome.

In addition descriptive statistics will be presented by means and standard deviations, as appropriate. When data are skewed, medians and quartiles will be calculated and for categorical data, frequencies and percentages will be presented.

Data will be analyzed using the data analysis software package IBM SPSS Statistics (version 19).

## Discussion

Acknowledging that dual tasking forms an integral part of daily functioning, the DUALITY study proposed here aims to investigate the efficacy and safety of a rehabilitation intervention to improve dual tasking in PD patients. We will establish robust evidence on which training modality, i.e. integrated or consecutive training, leads to the best training results and how this compares with a control period without intervention. Given the reduced capacity for consolidation of motor learning in PD [49,73] and the presence of executive deficits [28], it will be established whether dual task learning effects transfer to improvements in daily life and are sustained for 12 weeks without training.

A paucity of dual task studies have been performed in PD patients, most of which with poor methodological designs. This pilot work has shown short-term positive effects on gait performance [42-45], although there was considerable variation in the population, training period and tasks (motor, cognitive) that were studied. This is the first power-based and randomized trial that compares two interventions in an early to mid-stage PD population that are both aimed at improving dual task performance but with contrasting treatment strategies. In analogy with a DT training study in balance-impaired older people [51,52], we expect that both groups will show some improvement on DT outcomes However, as integrated DT training is more cognitively challenging we hypothesize that this will result in a better consolidation and retention of the training effects in line with motor learning theory [74]. In addition, we expect that integrated training will resort in better DT outcomes as the exact practice conditions mimics real life tasks more closely and this is important for transfer of learning [74]. In this study, the evaluation of motor performance will be supplemented by assessment of cognitive performance which allows monitoring of learning effects in both motor and cognitive domains. We focus on assessing various components of executive functions, an area that is specifically shown to be affected in PD patients [28]. This implies that this trial will also provide novel evidence on the impact of cognitive training on executive function in PD which may inform future power-based studies in this area.

This will be the first dual task training study that will look at differences in dual task gains between freezers versus non-freezers after stratification for these subgroups. The results of this study will establish which training strategy is optimal in each subgroup, taking their different cognitive profiles into account. Since freezers show greater executive deficits than their non-freezing counterparts [31-33,75] and learn less well in dual task conditions [34], we anticipate that freezers may benefit more from consecutive training in order to achieve dual task learning.

In conclusion, in this DUALITY trial we will study two different strategies aimed at improving dual tasking in PD patients. We expect that the trial will provide novel and clinically important information on the most effective and safe training strategy in different patient subgroups and in this way will contribute to developing future directions for rehabilitation targets in PD.

### Consent

Written informed consent will be obtained from every patient included in the study. A copy of the written consent will be available for review by the Editor of this journal.

## Abbreviations

PD: Parkinson’s disease; FOG: Freezing of gait; DT: Dual task; CTT: Consecutive task training; IDT: Integrated dual task training; RUMC: Radboud University Medical Centre; MMSE: Mini mental status examination; DBS: Deep brain stimulation; SD: Standard deviation; MoCA: Montreal cognitive assessment; FAB: Frontal assessment battery; UPDRS: Unified Parkinson’s disease rating scale; New FOGQ: New freezing of gait questionnaire; ABC: Activities specific balance confidence scale; ScopaCog: Scales for outcomes of Parkinson’s disease - cognition; ANT: Alternating names test; AIT: Alternating intake test; PDQ-39: Parkinson’s disease questionnaire for quality of life; ADL: Activities of daily living; MPT: Mobile phone task.

## Competing interests

The authors declare that they have no competing interests.

## Authors’ contributions

AN and MM conceived the idea for the study and obtained funding for the study. AN, SK and MM contributed to the research design. EM, CS, LM, WV and BB were involved in patient recruitment. AN, EM and CS were principally responsible for the drafting of the manuscript. All authors contributed to the design of the study, intervention and outcome measures. All authors assisted in editing the final submitted manuscript. All authors have read and approved the manuscript.

## Pre-publication history

The pre-publication history for this paper can be accessed here:

http://www.biomedcentral.com/1471-2377/14/61/prepub
